# Role of the PERK-eIF2*α*-CHOP Signaling Pathway in the Effect of Needle Knife Therapy on Knee Joint Chondrocyte Apoptosis

**DOI:** 10.1155/2019/7164916

**Published:** 2019-06-16

**Authors:** Yong-hui Yang, Tian-hao Liu, Li-da Zhang, Zhu-yue Chen, Xiao-shuang Huang

**Affiliations:** ^1^The Third Affiliated Hospital of Anhui University of Traditional Chinese Medicine, 45 Shihe Road, Hefei 230061, Anhui, China; ^2^Chinese Medicine College, Jinan University, 601 Huangpu West Avenue, Guangzhou 510632, Guangdong, China; ^3^The Graduate School, Anhui University of Traditional Chinese Medicine, 103 Meishan Road, Hefei 230038, Anhui, China

## Abstract

Needle knife therapy, a form of acupuncture and moxibustion, has been widely used in the clinical treatment of knee osteoarthritis (KOA). However, the mechanism is not clear. Therefore, we studied the mechanisms of action of needle knife intervention on KOA in rabbits, with the PERK-eIF2*α*-CHOP pathway as a starting point, in order to determine the mechanism underlying knee joint chondrocyte apoptosis. Apoptosis and ultrastructural changes in the articular cartilage were examined by pathological study and transmission electron microscopy, and PERK, eIF2*α*, and CHOP mRNA and protein levels were detected by qRT-PCR and western blot, respectively. PERK, eIF2*α*, and CHOP protein levels were significantly higher in the model group than in the normal group (*P* < 0.01) and were considerably downregulated in the needle knife and the medicine groups compared to the model group (*P* < 0.01). The eIF2*α*, p-eIF2*α*, and CHOP protein levels were not significantly different between the needle knife and medicine groups. The PERK, eIF2*α*, and CHOP mRNA levels in the drug group were higher than those in the needle knife group (*P* < 0.01). Needle knife therapy can regulate PERK-eIF2*α*-CHOP signaling pathway, which could be one of the mechanisms by which it affects chondrocyte apoptosis in KOA rabbits.

## 1. Introduction

Knee osteoarthritis (KOA) is a chronic degenerative joint disease characterized by degeneration of joint cartilage. KOA is associated with joint pain, swelling, deformity, and movement disorders. The pathogenesis of the disease has not been completely established, and no consensus has been reached on its clinical treatment, which often results in emotional and economic burdens to patients. Acupuncture and moxibustion have shown clear therapeutic effects on KOA and other diseases. Needle knife therapy, a form of acupuncture and moxibustion, has shown favorable effects in the clinical treatment of KOA [1–3]. There have been several studies on the mechanisms underlying the effects of needle knife therapy, including the effects of needle knife intervention on the biomechanics of the patellar ligament [4, 5], matrix metalloproteinases, extracellular matrix, and inflammatory factors [6–9].

Chondrocyte apoptosis plays an important role in regulating cartilage degeneration in KOA. However, there have been few studies on the effect of needle knife therapy on chondrocyte apoptosis in KOA. Endoplasmic reticulum stress (ERS) is one of the causes of chondrocyte apoptosis [10–12]. The relationship between ERS and the clinical efficacy and mechanism of action of needle knife therapy to treat KOA remains unclear. Further, the effect of needle knife therapy, if any, on improving or inhibiting chondrocyte apoptosis has not been determined to date.

The PERK-eIF2*α*-CHOP pathway is a key ERS pathway and was therefore used as a focal point in this study. Based on the Videman method, a KOA model in rabbits was established by stretching the right hind limb of the rabbit and fixing it at the immobilization position [13]. Pathology, electron microscopy, quantitative real-time PCR (qRT-PCR), western blot, and other methods were used to observe the effects of needle knife therapy and diclofenac diethylamine emulsion on the apoptosis and ultrastructure of knee joint chondrocytes and the mRNA and protein expression of PERK, eIF2*α*, and CHOP in these chondrocytes, and the underlying mechanism of action was explored. The aim of this study was to determine the mechanism of action of needle knife therapy on chondrocyte apoptosis in KOA and thereby provide a scientific basis for elucidating the clinical efficacy and mechanism of action of needle knife therapy for KOA.

## 2. Materials and Methods

### 2.1. Experimental Animals and Grouping

Forty New Zealand white rabbits aged 5 months, with a body weight of 2.0–2.5 kg, were used in the study. These rabbits were provided by Pizhou Dongfang Breeding Co., Ltd. in Jiangsu, China (SCXK (Su) 2014-0005). The experiment was approved by the Ethics Committee of Anhui University of Chinese Medicine and implemented in strict accordance with the requirements of the Ethics Committee throughout the process. Conventional feeding: Animals were given access to water and food* ad libitum* at a room temperature of 20–22°C and a humidity of 40%–60% under 12/12 h alternating cycles of light and dark. The animals were included in experiments after 3 days of conventional feeding.

The animals were randomly divided into the normal, model, needle knife, and medicine groups, with 10 animals in each group. KOA model was established in all the groups except the normal group. Two rabbits in each group were used for pathological tests for confirming successful modeling, and the remaining rabbits were used for testing of other indices.

### 2.2. Reagents

Resin bandage for fixing (15 cm × 180 cm, Zhuhai Lizhu Medical Biomaterial Co., Ltd.) and needle knife (0.4 mm × 40 mm, Beijing Zhuoyue Huayou Medical Devices Co., Ltd.) were used in the study. Primer synthesis was performed by Sangon Biotech, and the following reagents were used: Trizol (Life Technologies); SDS kit (Sigma); ECL hypersensitive luminescence kit (Thermo Fisher Scientific, USA); SDS-PAGE gel preparation kit (Beyotime), DAB chromogenic agent (ZLI-9018), PBS (ZLI-9062), and EDTA repair solution (ZLI-9067; Beijing Zhongshan Golden Bridge Biotechnology Co., Ltd., China); hematoxylin (BA-4041; Baso Diagnostic Inc., Zhuhai, China); RIPA cell lysis buffer, western primary and secondary antibody dilution buffer (Beyotime, China); PVDF membrane and Tris base (Solarbio, USA); *β*-Actin (Zs-BIO); PERK, eIF2*α*, and CHOP kit (Bioworld); p-PERK kit (Bioss); p-eIF2*α* kit (CST).

### 2.3. Establishment of KOA Model in Rabbits and Model Evaluation

Before modeling, the rabbits were fasted for 12-16 hours and anesthetized by ear vein injection of 3% sodium pentobarbital solution (30 mg/kg). Based on the classic Videman method [13], the rabbits' right hind limbs were stretched and fixed by plaster for 5 weeks. The rabbits were fixed from the groin to the ankle (with the knee joint stretched straight at 180°); the toes were exposed for observation of blood supply; if the toes appeared necrotic, the plaster bandage was immediately removed and was not refixed until the swelling disappeared. The plaster bandage was refixed if it was loosened or peeled off.

**Figure 1:**
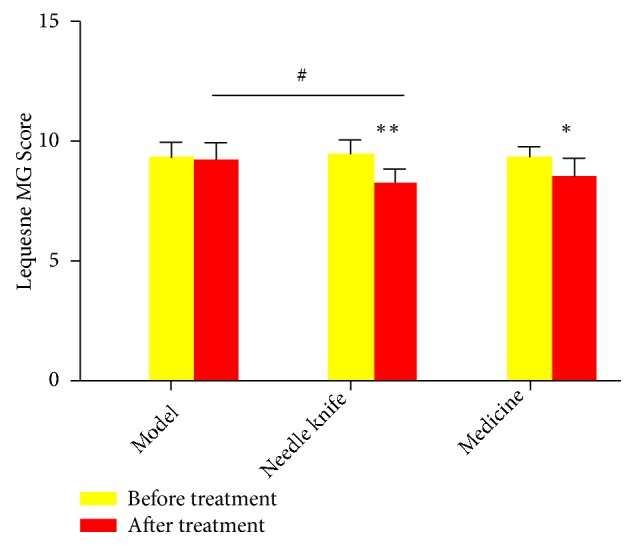
*Evaluation of knee joint activity in each group by using Lequesne MG scores*. ^*∗*^*P* < 0.05, ^*∗∗*^*P* < 0.01 compared with the same group before treatment; ^#^*P* < 0.05, ^##^*P* < 0.01 compared with the model group after treatment.

According to the modified Lequesne index of severity for osteoarthritis of the knee [14], behavioral assessments were performed in each group of animals after modeling and after treatment. Two rabbits from each group were randomly selected for pathological tests, and the structure of the knee joint cartilage was observed under an optical microscope (Figure 1).

### 2.4. Intervention and Treatment

The rabbits in the normal group were conventionally fed and the rabbits in the model group were conventionally fed after modeling. After modeling, the middle point of the patellar ligament and the medial/lateral collateral ligament from the left knee joint of the rabbit in the needle knife group was taken as the insertion point for a needle knife and marked with a surgical marking pen. After routine skin preparation and disinfection, needle knife therapy was performed as follows: (1) Loosening the patellar ligament: the knife was rapidly inserted with the edge line in parallel with the patellar ligament and perpendicular to the skin. After reaching the patellar ligament, the needle knife head was rotated at 90° to be perpendicular to the patellar ligament, and incising was repeated rapidly 3–5 times. After removal of the knife, the incision was pressed with sterile gauze for hemostasis. (2) Loosening the medial and lateral collateral ligaments: the knife was rapidly inserted with the edge line in parallel with the medial collateral ligament and perpendicular to the skin. After reaching the patellar ligament, the needle knife head was rotated at 90° to be perpendicular to the patellar ligament, and incising was repeated rapidly 3–5 times. After removal of the knife, the incision was pressed with sterile gauze for hemostasis. The lateral collateral ligament was loosened using the same method as for the medial collateral ligament. The treatment was administered once a week for 3 weeks. After modeling, diclofenac diethylamine emulsion (Beijing Novartis Pharma Co., Ltd., batch number: VP1735) was locally applied to the affected knee of the rabbits in the medicine group twice a day for 3 weeks.

### 2.5. Test Indices

#### 2.5.1. HE Staining

Two rabbits were randomly selected from each group and were anesthetized by intraperitoneal injection of 0.3% sodium pentobarbital (30 mg/kg); cartilage from the tibial plateau and the lower femoral condyle of the right knee joint were cut off with a sharp scalpel on an ice tray. A piece of fresh cartilage tissue 2 cm × 2 cm in size was rapidly cut with a scalpel from the medial femoral condyle and the lateral femoral condyle, respectively; parts of the specimens were fixed in 10% formalin solution and sliced after HE staining. In each group, 2-3 fields were selected from 3-5 sections for observation of the morphology of the cartilage by using an optical microscope.

#### 2.5.2. Transmission Electron Microscopy (TEM)

Several 3 mm × 3 mm × 3 mm cartilage tissue blocks were isolated from another part of the specimens, rinsed, fixed with osmic acid, dehydrated, and embedded according to the conventional specimen preparation procedure for TEM. Four to six ultrathin 60-nm-thick sections were made for each animal in each group. After uranyl acetate-lead citrate staining, 3-5 fields were randomly selected from each section for observation and photography of the structure and morphology of chondrocytes by using a JEM1230 TEM.

Eight rabbits were randomly selected from each group, and the cartilage from the tibial plateau and the lower femoral condyle of the right knee joint were cut off using the method described earlier and stored at -80°C for western blot and qRT-PCR assays.

#### 2.5.3. Western Blot

Eight rabbits were randomly selected from each group; the cartilages from the tibial plateau, and the lower femoral condyle of the right knee joint were cut off as described previously and stored at -80°C for western blot assay. Protein levels of PERK, eIF2*α*, and CHOP in the knee joint cartilage were determined by western blot assay as follows: the cartilage was added to RIPA cell lysis buffer and ground. The supernatant was removed and centrifuged at 4°C and 13684 ×g for 15 min, total protein was extracted from the tissue sample, and the protein concentration was measured by BCA Protein Assay Kit. ImageJ was used to analyze the gray values of the bands in the fluorescence imaging, and the relative expression level of a protein was determined as the ratio of the expression level of the protein to that of the internal standard protein, *β*-actin.

#### 2.5.4. Quantitative Real-Time PCR

The relative mRNA expression levels of PERK, eIF2*α*, and CHOP in the knee joint cartilage were determined by qRT-PCR. The tissue was ground with liquid nitrogen, and total RNA was extracted from the tissue sample by using TRIzol. After nucleic acid gel electrophoresis, each of the RNAs was determined by ultraviolet spectrophotometry. The content and purity of RNAs were calculated, and reverse transcription was carried out using a reverse transcription kit, to obtain a reverse transcription product cDNA from approximately 3 *μ*g of RNA. Then PCR was performed. The mRNA CT values of PERK, eIF2*α*, and CHOP were obtained at the end of the reaction, and the relative expression levels of the target genes were calculated by the 2-ΔΔCT method. Primer sequences and product lengths are shown in Table 1.

### 2.6. Statistical Methods

Statistical analyses were performed using SPSS 18.0. All the experimental data were expressed as mean ± standard deviation ($\overset{-}{x}\pm s$). For comparison of numerical variables between groups, if the variables conformed to the normal distribution with homogeneous variance, the intergroup differences were evaluated using the least significant difference method (LSD) following the one-way ANOVA; otherwise, the differences were evaluated using the Kruskal-Wallis* H* rank sum test. For the intragroup comparison of variables before and after treatment, if the variables conformed to the normal distribution, they were evaluated using the paired samples* t*-test; otherwise, the signed rank sum test was used.* P *< 0.05 was considered statistically significant. Statistical graphs were drawn using GraphPad Prism 7.0.

## 3. Results

### 3.1. Effect of Needle Knife Therapy on Knee Joint Activity Evaluated by Lequesne MG Score

As shown in Figure 1, there was no significant change in knee joint activity score in the model group after treatment. The needle knife and medicine groups had significantly reduced knee joint activity scores compared to those of the model group (*P* < 0.01).

### 3.2. Effect of Needle Knife Therapy on Knee Joint Chondrocytes

**Figure 2:**
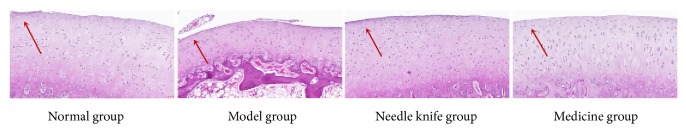
*Comparison of knee joint chondrocytes in the study groups (×20)*. The knee joints of rabbits in the needle knife group and medicine group had thicker cartilage layers, more chondrocytes, and relatively clear subchondral tidemarks compared with the model group.

As shown in Figure 2, the knee joints of rabbits in the normal group had evenly distributed chondrocytes and bone cells in the cartilage matrix; the cartilage layer was relatively thick, with several chondrocytes and a clear subchondral tidemark. The knee joints of rabbits in the model group had relatively thin and clear cartilage layers with fewer chondrocytes; the surface layer of the cartilage was fibrotic, and the chondrocytes in each layer proliferated with slight osteofibrosis in some chondrocytes; the cartilage layer was significantly different from that of the normal group. Compared with the model group, the needle knife and medicine groups had thicker cartilage layers, more chondrocytes, and relatively clear subchondral tidemarks.

### 3.3. Effect of Needle Knife Therapy on Knee Joint Chondrocyte Ultrastructure

**Figure 3:**
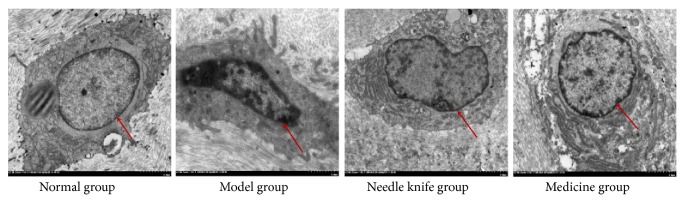
*Comparison of knee joint chondrocyte ultrastructure in the study groups*. The chondrocytes from the needle knife group and medicine group had slightly folded nuclear membranes with intact membrane structure compared with the model group.

The knee joint chondrocytes of rabbits in the normal group had smooth nuclear membranes; nuclear membrane spaces were not dilated, chromatins in the nuclei were homogeneous, and the organelles were normal, as revealed by TEM. The chondrocytes in the model group had slightly dilated nuclear membranes with absence of part of the nuclear membranes; chromatins aggregated in the nuclei with obvious clustering around the borders, nuclei were slightly pyknotic, rough endoplasmic reticula were broken and reduced, and mitochondria showed obvious vacuolar degeneration. The chondrocytes from the needle knife and medicine groups had slightly folded nuclear membranes with intact membrane structure; the chromatins in the nuclei were slightly aggregated with unremarkable clustering around the borders, and broken rough endoplasmic reticula and vacuolar degeneration in few mitochondria were observed in the cytoplasm (Figure 3).

### 3.4. Needle Knife Therapy Downregulated the Expression of PERK and p-PERK Proteins in the Knee Joint Cartilage

**Figure 4:**
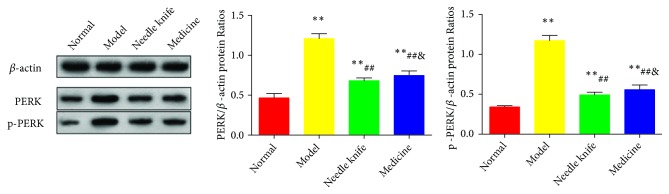
*Comparison of PERK and p-PERK protein levels in the knee joint cartilage of the study groups (n = 8)*. ^*∗*^*P* < 0.05, ^*∗∗*^*P* < 0.01 compared with the normal group; ^#^*P* < 0.05, ^##^*P* < 0.01 compared with the model group; ^&^*P* < 0.05, ^&&^*P* < 0.01 compared with the needle knife group.

As shown in Figure 4, the normal group showed significantly lower levels of PERK and p-PERK protein in the knee joint cartilage than the model, needle knife, and medicine groups did (*P* < 0.01). The needle knife and medicine groups had significantly lower levels of PERK and p-PERK proteins than the model group did (*P* < 0.01). The needle knife group had significantly lower levels of PERK and p-PERK proteins than the medicine group did.

### 3.5. Needle Knife Therapy Downregulated eIF2*α*, p-eIF2*α*, and CHOP Protein Expression in the Knee Joint Cartilage

**Figure 5:**
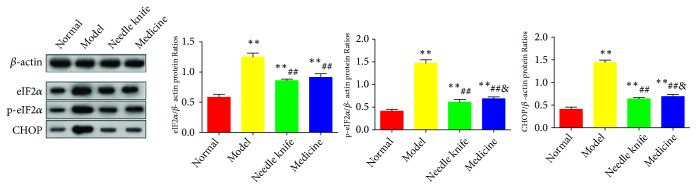
*Comparison of eIF2α and CHOP protein levels in the knee joint cartilage of the study groups (n = 8)*. ^*∗*^*P* < 0.05, ^*∗∗*^*P* < 0.01 compared with the normal group; ^#^*P* < 0.05, ^##^*P* < 0.01 compared with the model group; ^&^*P* < 0.05, ^&&^*P* < 0.01 compared with the needle knife group.

The levels of eIF2*α*, p-eIF2*α*, and CHOP proteins were significantly higher in the model group than in the normal group (*P* < 0.01). The needle knife group had significantly lower protein levels of eIF2*α*, p-eIF2*α*, and CHOP than the model group did (*P* < 0.01). There were no significant differences in the protein levels of eIF2*α*, p-eIF2*α*, and CHOP between the needle knife and medicine groups (Figure 5).

### 3.6. Needle Knife Therapy Downregulated the mRNA Expression of PERK, eIF2*α*, and CHOP in the Knee Joint Cartilage

**Figure 6:**
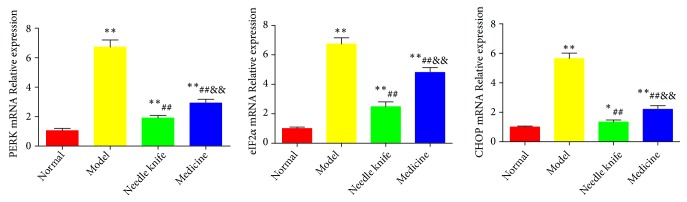
*Comparison of PERK, eIF2α, and CHOP mRNA levels in the knee joint cartilage of the study groups (n = 8)*. ^*∗*^*P* < 0.05, ^*∗∗*^*P* < 0.01 compared with the normal group; ^#^*P* < 0.05, ^##^*P* < 0.01 compared with the model group; ^&^*P* < 0.05, ^&&^*P* < 0.01 compared with the needle knife group.

As shown in Figure 6, the levels of PERK, eIF2*α*, and CHOP mRNA in the knee joint cartilage were significantly lower in the normal group than in the model group (*P* < 0.01). The needle knife and medicine groups had significantly lower mRNA levels of PERK, eIF2*α*, and CHOP than the model group did (*P* < 0.01). The medicine group had significantly higher PERK, eIF2*α*, and CHOP mRNA levels than the needle knife group did (*P* < 0.01).

## 4. Discussion

KOA is a chronic bone and joint disease involving abnormal reconstruction of joint cartilage, caused by unbalanced coupling between synthesis and degradation of chondrocytes, cartilage matrix, and subchondral bones due to interactions between biological and mechanical factors. With increasing understanding of apoptosis, the development and progression of KOA have been linked to chondrocyte apoptosis [15].

In this study, we found that chondrocyte apoptosis was significantly higher in the KOA model group than in the normal group and was accompanied by pathological and morphological changes, indicating joint chondrocyte apoptosis in KOA. The joint chondrocyte apoptosis could be attributed to the following process: fixation of the knee joint at stretching position limited joint movement. The persistent contraction of the muscles and joint sacs across the joints increased pressure on the joint surface, and the components of mucin and hyaluronic acid decreased under the abnormal stress state. This resulted in mechanical wear and tear of the joint surface, adhesion between the synovial membrane and cartilage, and compromised nutrient uptake by the chondrocytes, causing microenvironmental changes within the joint and leading to excessive chondrocyte apoptosis. After needle knife therapy, joint chondrocyte apoptosis decreased, the cartilage layer thickened, and the number of chondrocytes increased significantly in the KOA model. Thus, needle knife therapy could ameliorate the stress inside and outside the joint by loosening the major ligaments around the knee joint and reducing the undermining cartilage pressure exerted on the joint. The metabolism of chondrocytes and the joint mechanical balance were thus improved, and the stability of the microenvironment within the joint was regulated. Consequently, chondrocyte apoptosis was inhibited and joint cartilage degeneration was alleviated [14–17].

ERS plays a key regulatory role in the initiation of chondrocyte apoptosis by activating the unfolded protein response (UPR) [18–20]. The UPR includes three signaling pathways, including the PERK pathway. The expression levels of the ERS markers PERK and CHOP increase during the progression of osteoarthritis; CHOP can regulate chondrocyte apoptosis, and CHOP-mediated chondrocyte apoptosis accelerates joint cartilage degeneration [21, 22]. This study showed that the rabbit model of KOA had increased PERK, p-PERK, eIF2*α*, p-eIF2*α*, and CHOP mRNA and protein expression in knee joint chondrocytes. The following process likely occurred in the KOA model: As the microenvironment within the knee joint changed, PERK was activated by phosphorylation. Activated PERK phosphorylated eukaryotic translation initiation factor 2 alpha (eIF2*α*) and activated the expression of its target gene C/EBP homologous protein (CHOP) and the ERS response, thereby initiating apoptosis process, inducing chondrocyte apoptosis, and accelerating joint cartilage degeneration [21–23]. Needle knife therapy could normalize the microenvironment within the knee joint and reduce mRNA and protein levels of PERK, p-PERK, eIF2*α*, p-eIF2*α*, and CHOP, which facilitated the inhibition of the ERS response in the knee joint, inhibiting the initiation of the PERK-eIF2*α*-CHOP signaling pathway in the UPR during chondrocyte apoptosis and preventing and ameliorating joint cartilage degeneration.

In addition, this study showed that diclofenac diethylamine emulsion alleviated acute and chronic inflammatory reactions and reduced inflammatory swelling and pain, but was not as effective as needle knife therapy in regulating the PERK-eIF2*α*-CHOP signaling pathway in KOA chondrocytes and maintaining the microenvironmental homeostasis within the knee joint. By loosening the spasm of soft tissue around the knee joint, needle knife therapy could relieve pain, adjust the mechanical balance between the knee joint ligaments, and mediate biological changes in the cartilage tissue, which had a protective effect in repairing joint cartilage and delaying cartilage degeneration and joint damage.

In this study, ERS was taken as a starting point. The regulation of the PERK-eIF2*α*-CHOP signaling pathway by needle knife therapy could be one target mechanism to interfere with the apoptosis of knee joint chondrocytes, which provides a scientific basis for the use of needle knife therapy in the treatment of KOA. ERS-induced apoptosis is a multichannel-mediated combined effect [24, 25]. Only part of the entire mechanism is explored by investigating the mechanism by which needle knife therapy inhibits knee joint chondrocyte apoptosis in KOA rabbits and facilitates the histomorphological recovery of the joint cartilage via the PERK-eIF2*α*-CHOP signaling pathway in the UPR. The complete pathway by which needle knife therapy interferes with chondrocyte apoptosis remains unclear and merits further research.

## 5. Conclusion

By studying the effect of needle knife therapy on knee joint chondrocyte apoptosis in a rabbit model of KOA, we found that regulation of the PERK-eIF2*α*-CHOP signaling pathway by needle knife therapy could interfere with apoptosis of knee joint chondrocytes. Our findings provide a scientific basis for the use of needle knife therapy in the treatment of KOA.

## Figures and Tables

**Table 1:** Primer sequences of target genes for RT-PCR.

Primer name		Primer sequence (5′ – 3′)	Amplification length (bp)
*β*-actin	Forward	CAGCCCTCCTTCATCGGTAT	113
	Reverse	GACATGACGTTGTTGGCGTA	
PERK	Forward	GCGGCAATGAGAAGTGGAAT	108
	Reverse	TCCCTCTGGGCTTAAAGGTG	
eIF2*α*	Forward	CTCCTGAAAGCAGCAACCTC	128
	Reverse	GACCGAGATGAAGCATCGTG	
CHOP	Forward	CTTCCATGTAGCGGAGTCCT	142
	Reverse	GTGAGAGCCAGTCTCCCTTT	

Primer sequences of target genes for RT-PCR and all values of amplification length (bp) are listed.

## Data Availability

The data used to support the findings of this study are available from the corresponding author upon request.
